# Antibacterial Effect of High-Purity Nisin Alone and in Combination with D-Amino Acids or Chlorhexidine in an Endodontic-Like Biofilm Model

**DOI:** 10.3390/antibiotics10020149

**Published:** 2021-02-02

**Authors:** Ericka T. Pinheiro, Lamprini Karygianni, Thomas Attin, Thomas Thurnheer

**Affiliations:** 1 Department of Dentistry, School of Dentistry, University of São Paulo, São Paulo 01000-000, Brazil; 2 Clinic of Conservative and Preventive Dentistry, Center of Dental Medicine, University of Zurich, CH-8032 Zürich, Switzerland; lamprini.karygianni@zzm.uzh.ch (L.K.); thomas.attin@zzm.uzh.ch (T.A.); thomas.thurnheer@zzm.uzh.ch (T.T.)

**Keywords:** nisin, D-amino acids, chlorhexidine, biofilm, endodontic pathogens, endodontic-like multispecies biofilm

## Abstract

New strategies to eradicate endodontic biofilms are needed. Therefore, we evaluated the effect of high-purity nisin alone and in combination with D-amino acids (D-AAs) or chlorhexidine (CHX) against an “endodontic-like” biofilm model. Biofilms were grown on hydroxyapatite discs for 64 h and treated with nisin, eight D-AAs mixture, nisin + eight D-AAs, 2% CHX, and nisin + 2% CHX. After the 5 min and 24 h treatments, biofilm cells were harvested and total colony-forming units were counted. Differences between groups were tested by two-way ANOVA followed by Tukey’s multiple comparisons test (*p* < 0.05). Nisin and D-AAs, alone or in combination, were not effective in reducing bacteria after short or long exposure times. After 5 min, treatment with 2% CHX and nisin + 2% CHX resulted in 2 and 2.4-log cell reduction, respectively, compared with the no treatment control (*p* < 0.001). After 24 h, 2% CHX and nisin + 2% CHX drastically reduced bacterial counts. In conclusion, high-purity nisin alone or in combination with D-AAs did not show antibacterial activity against multispecies biofilms. Moreover, combined treatment using nisin and CHX showed similar antibiofilm activity compared with the use of CHX alone.

## 1. Introduction

Apical periodontitis is a biofilm-induced disease usually detected in teeth with pulp necrosis or failed root canal treatment [1]. Intraradicular biofilms are formed by polymicrobial communities, which are attached to the root canal dentin and embedded in an extracellular matrix [1]. The mechanical action of endodontic instruments along with irrigation represents the main strategy for the disruption and removal of intraradicular biofilms [2]. However, residual bacteria in untouched areas of the root canal are difficult to eliminate as they can resist the action of antimicrobials commonly used for root canal treatment [3,4,5,6]. Therefore, there is a demand for new strategies to eradicate biofilms established in the root canals of teeth with apical periodontitis.

A variety of promising antibiofilm agents has been studied over the years, including the antimicrobial peptides. Nisin is a natural antimicrobial peptide produced by lactic acid bacteria and is one of the most studied bacteriocin so far [7,8,9,10]. Nisin can rapidly penetrate biofilms and form pores in the membrane cells [11,12]. It has been proven to be effective against single-species biofilms of Gram-positive organisms found in catheters, wounds, and food [9,11,12]. Similarly, nisin has shown promising results against oral pathogens [7,10], including those involved in root canal infections [13,14,15]. Indeed, the high purity form of nisin (>95% purity) was found to be more potent than the low content nisin (2.5% purity) against multispecies oral biofilms [7]. As high-purity nisin presents antibiofilm activity with low levels of cytotoxicity to human cells, it has been considered a potential therapeutic agent to prevent or treat oral diseases [7].

Other antibiofilm strategy includes the use of D-amino acids (D-AAs), which can interfere with the cell wall synthesis and promote biofilm dispersal [16,17]. As disperser cells are generally more susceptible to antimicrobial treatment than the unbroken biofilm, the association of D-AAs to other agents has been considered a useful strategy to treat pre-established biofilms. Indeed, a previous study demonstrated the synergistic effect of a mixture of three D-AAs (D-cysteine, D-aspartic acid, and D-glutamine) in combination with nisin against *Streptococcus mutans* biofilms [18]. Recently, the efficacy of a mixture of eight D-AAs (D-methionine, D-leucine, D-tyrosine, D-tryptophan, D-serine, D-threonine, D-phenylalanine, and D-valine) was found to be better than that of four D-AAs against multispecies biofilms in cooling water systems [19].

In endodontics, antibiofilm agents may have potential application for root canal irrigation (short-term treatment) and inter-appointment medication (long-term treatment). High-purity nisin has been suggested as a potential alternative to traditional disinfecting agents used for root canal irrigation due to its ability to disrupt *Enterococcus faecalis* biofilms when applied for 10 min [15]. However, the long-term effect of high-purity nisin and the synergistic effect of nisin and a mixture of 8 D-AAs against endodontic biofilms have not yet been explored. Therefore, the objective of this study was to evaluate the short- and long-term antibacterial effect of high-purity nisin alone or in combination with eight D-AAs using an eleven-species biofilm model (Zurich “endodontic-like” biofilm model) [20,21,22]. Additionally, the antibiofilm activity of nisin in combination with chlorhexidine was investigated.

## 2. Results

**Figure 1:**
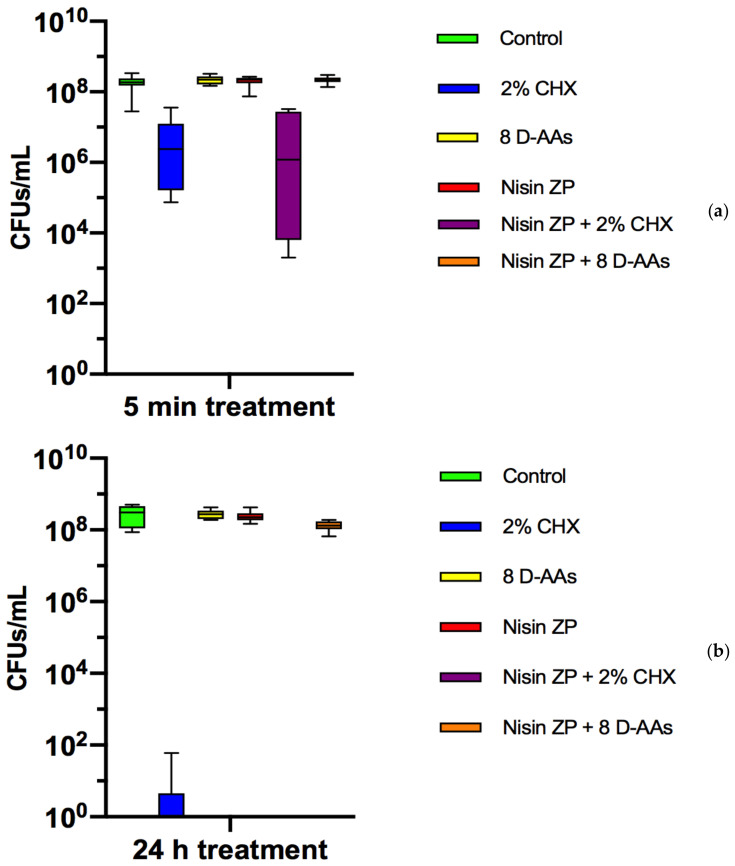
Boxplots demonstrating cell counts after 5 min (**a**) and 24 h (**b**) treatments with 2% chlorhexidine (CHX), a mixture of eight D-amino acids (D-AAs), high-purity nisin (nisin ZP), and their associations on multispecies biofilms grown on hydroxyapatite disks for 64 h. The internal line represents the median; the whiskers indicate minimum and maximum. Data derive from three independent experiments, each represented in triplicate biofilm cultures (*n* = 9).

Preformed biofilms were treated with 2% CHX, mixture of eight D-AAs, nisin, nisin + 2% CHX, and nisin + 8 D-AAs. The colony-forming units (CFUs) counts in the biofilms with and without treatment (control) are shown in Figure 1. After 5 min, no reduction of bacterial cells was observed with nisin and eight D-AAs treatments, alone or in combination, when compared with the control (8.2log_10_ CFU/mL ± 0.3). In contrast, the treatment of preformed biofilms with 2% CHX within 5 min (6.2log_10_ CFU/mL ± 0.1) achieved 2-log cell reduction compared with the control (*p* < 0.0001). The combined treatment of nisin with 2% CHX within 5 min (5.8log_10_ CFU/mL ± 1.7) showed no difference when compared with CHX treatment alone (*p* > 0.05). After 24 h, 2% CHX and nisin + 2% CHX showed the best antibacterial activity among all treatments.

**Figure 2:**
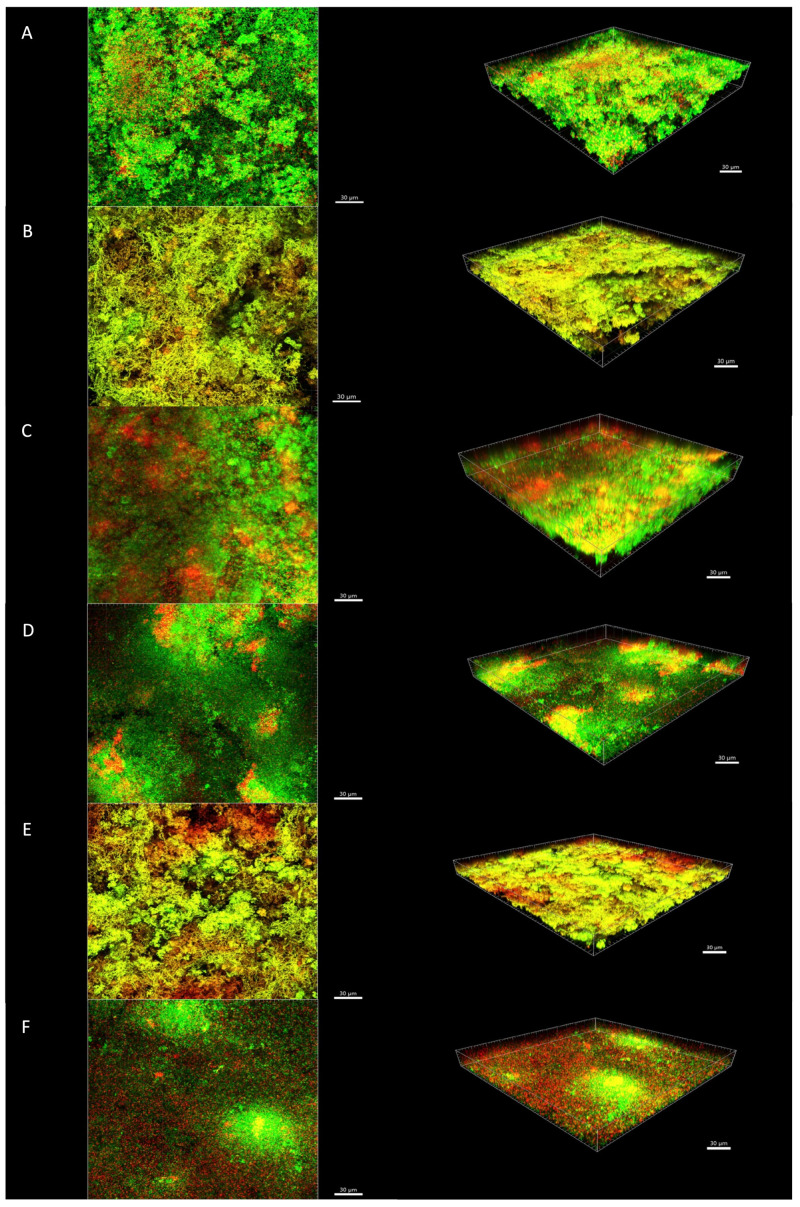
Confocal laser scanning microscopy images of in vitro biofilms after 5-min treatment. (**A**) Control group with no treatment; (**B**) 2% CHX; (**C**) eight D-AAs; (**D**) high-purity nisin; (**E**) high-purity nisin + 2% CHX; and (**F**) high-purity nisin + eight D-AAs. The biofilms were stained using the LIVE/DEAD Viability Kit; live cells appear green and dead cells red. Images represent the 3D reconstructions of the biofilms using the IMARIS 7.6.5 software (orthogonal and perspective views from left to right). Scale bar 30 μm.

**Figure 3:**
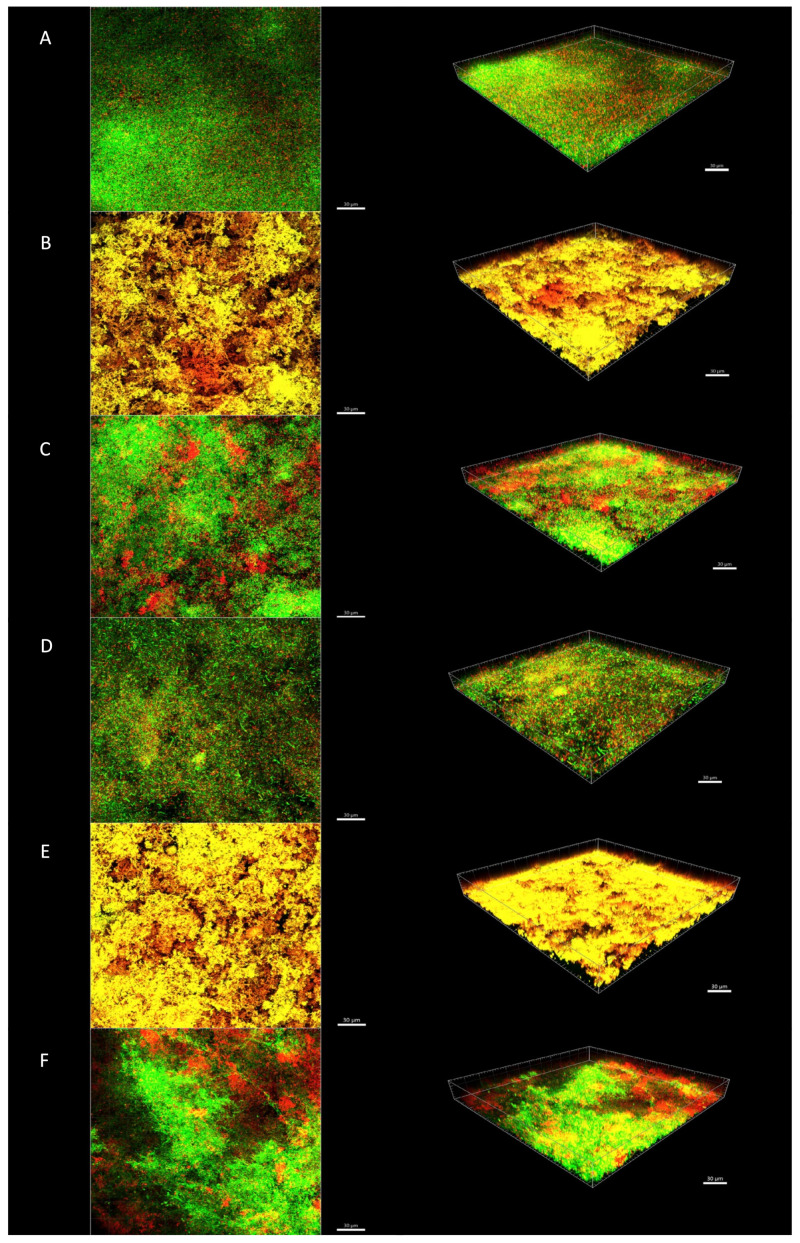
Confocal laser scanning microscopy images of in vitro biofilms after 24 h treatment. (**A**) Control group with no treatment; (**B**) 2% CHX; (**C**) eight D-AAs; (**D**) high-purity nisin; (**E**) high-purity nisin + 2% CHX; and (**F**) high-purity nisin + eight D-AAs. The biofilms were stained using the LIVE/DEAD Viability Kit; live cells appear green and dead cells red. Images represent the 3D reconstructions of the biofilms using the IMARIS 7.6.5 software (orthogonal and perspective views from left to right). Scale bar 30 μm.

Figure 2 and Figure 3 show an illustrative series of confocal laser scanning microscopy (CLSM) images of the biofilms after short- and long-term treatments, respectively. CLSM images after treatment with 2% CHX and nisin + 2% CHX showed the presence of orange/red cells distributed throughout the biofilm, especially in the 24-h treatment (Figure 3B,E). The untreated control discs showed a predominance of green cells, indicating bacterial viability (Figure 2A and Figure 3A). Some cells in the control discs were stained red, which indicates the presence of dead bacteria due to natural cell turnover during biofilm growth. Red cells could also be observed in CLSM images of biofilms treated with 8 D-AAs (Figure 2C and Figure 3C) and nisin (Figure 2D and Figure 3D). Their presence was even more pronounced after treatment with nisin + 8 D-AAs (Figure 2F and Figure 3F). Although these substances may have caused some damage to the cell membrane, they did not significantly affect the total CFU counts, suggesting that most bacteria remained viable after treatment.

## 3. Discussion

Nisin, a bacteriocin commonly used for food preservation, is considered a promising therapeutic agent for oral-biofilm related diseases [7,10]. The microbiological benefits of nisin have been investigated in the context of dental caries and periodontal disease as an agent to prevent biofilm formation [10]. In the context of endodontics, nisin has been tested alone or in combination with endodontic irrigants against biofilms of single species, especially of *E. faecalis* [14,15]. In order to better replicate biofilms that are found in endodontic infections, we used an eleven-species biofilm model to test the antimicrobial efficacy of a high content form of nisin (>95% purity). In this model, *E. faecalis* was incorporated into an established in vitro multispecies biofilm model, also known as “Zurich” biofilm model [20,21,22]. Under the studied conditions, no reduction in total bacterial counts was found after treatment of pre-formed biofilms with the high-purity nisin. These findings are in contrast with previous studies showing the activity of the aforementioned agent against saliva derived multispecies biofilms [7] or single-species biofilms [15]. The endodontic-like biofilm model used in the present study, which comprised mainly Gram-negative species, may have contributed to the poor activity of nisin. Previous studies have shown that the outer membrane of Gram-negative organisms may prevent nisin from reaching its target (lipid II) in the inner membrane [9]. Moreover, the integration of *E. faecalis* in the multispecies biofilms may have contributed to the high survival rate of bacteria after treatment. It has been shown that the presence of *E. faecalis* might favor the establishment of Gram-negative species in the biofilms [22,23]. However, as the residual bacteria were not identified in the present study, no conclusive remarks could be drawn about the susceptibility of specific species to nisin treatment.

A previous study showed that the association of nisin with a mixture of three D-AAs was able to increase the activity of nisin against biofilms of *Streptococcus mutans* [18]. The role of D-AAs in biofilm disassembly was first described by Kolodkin-Gal et al. [16], who showed that the four D-AAs treatment caused the release of amyloid fibers that linked cells in the biofilm together. Recently, it has been shown that D-AAs were able to eradicate *E. faecalis* biofilms by promoting biofilm disruption and reducing the number of viable cells [24,25]. In contrast, the eight D-AAs treatment alone or in association with the high-purity nisin did not reduce the total bacterial counts of endodontic-like biofilms in the present study. However, some red cells could be seen in the CLSM images, especially after treatment with the association of eight D-AAs and high purity nisin. Data from bacterial counts and CLSM images together suggest that non-lethal damage to cells may have occurred after treatment with nisin and D-AAs. Further work is needed to describe in detail the microscopic architecture of the biofilm after treatment with nisin and D-AAs. Moreover, as disrupted biofilms are expected to be more susceptible to antiseptics, sequential treatment with substances to disrupt biofilms and nisin should be tested in future studies.

In turn, 2% CHX alone or in combination with high-purity nisin were the best antibiofilm strategies tested, especially as long-term antibiofilm treatments. Chlorhexidine is a cationic bisbiguanide that binds with negatively charged surfaces of bacteria, causing damage in the bacterial cell wall and loss of cytoplasmatic components [26]. Due to the broad-spectrum of activity and the long-standing antibacterial effect on the root canal dentin, chlorhexidine has been used as an alternative antimicrobial substance in endodontics, usually at higher concentration (2%) than that found in mouthwash products [26]. Chlorhexidine was included in this study instead of sodium hypochlorite, which is the gold standard for endodontic irrigation, because it could be used for both short-term (endodontic irrigation) and long-term treatment (intracanal medication). In the 5-min treatment, 2% CH achieved only 2-log reduction of bacterial cells compared with the no-treatment control. This finding is in line with previous studies showing a limited action of chlorhexidine against established biofilms [3,6].

The combined use of 2% CHX and high-purity nisin did not improve chlorhexidine activity against oral biofilms. This finding is in contrast with previous studies showing that the combined treatment of nisin and endodontic irrigants increased the antibiofilm activity compared to the effects of irrigants when used alone. The endodontic irrigant MTAD (i.e., mixture of doxycycline, citric acid, and Tween 80) in combination with low-purity nisin showed better activity against *E. faecalis* biofilms compared with MTAD alone [14]. Similarly, the combination of high-purity nisin with low doses of sodium hypochlorite enhanced the activities of both antimicrobial agents against *E. faecalis* biofilms [15]. On the other hand, the combined use of nisin and chlorhexidine and CHX alone showed strong performance in killing bacterial cells within 24 h. The age of the biofilm in the present study (approximately 3 d) may have contributed to the high killing rate. Previous studies have shown that the proportion of killed bacteria after 2% CHX treatment was much lower in biofilms of 20 days or older than in young biofilms of 2–10 d [3,6]. Future studies using mature biofilms should be performed in order to evaluate the synergy effect of nisin and CHX when used as long-term treatment. In this context, testing different concentrations of nisin and CHX would be important to identify those that are synergistic.

## 4. Conclusions

The high-purity nisin alone or in combination with a mixture of eight D-AAs did not show antibacterial activity against preformed multispecies biofilms after short or long exposure times. Moreover, combined treatment using nisin and CHX showed similar antibiofilm activity compared with the use of CHX alone.

## 5. Materials and Methods

### 5.1. Biofilm Preparation

The in vitro biofilm model used in this study consisted of 11 bacterial species commonly found in endodontic infections and is a combination of models described earlier [20,21,22]. The biofilm contained *Actinomyces oris* OMZ 745, *Campylobacter rectus* OMZ 388 (ATCC 33238), *Enterococcus faecalis* OMZ 422 (ATCC 29212), *Fusobacterium nucleatum* OMZ 598 (KP-F2), *Parvimonas micra* OMZ 518 (ATCC 33270), *Porphyromonas gingivalis* OMZ 925 (ATCC 33277), *Prevotella intermedia* OMZ 278 (ATCC 25611), *Selenomonas sputigena* OMZ 527 (ATCC 35185), *Streptococcus oralis* OMZ 607 (SK 248), *Tannerella forsythia* OMZ 1132 (ATCC 43037), and *Veillonella dispar* OMZ 493 (ATCC 17748).

Biofilms were grown in 24-well polystyrene cell-culture plates on hydroxyapatite discs (9-mm diameter; Clarkson Chromatography Products, South Williamsport, PA, USA) that had been preconditioned in 1 mL of pasteurized and filter-sterilized saliva, and incubated for 4 h at room temperature [27]. The same saliva batch was used in all experiments. To initiate biofilm formation, the discs were covered with 1.6 mL of growth medium containing saliva and modified fluid universal medium (mFUM), and 200 μL of a microbial suspension prepared from equal volumes and densities of each strain, corresponding to an optical density at 550 nm of 1.0. The mFUM is a tryptone-yeast-based broth medium designated as FUM [28] and modified by supplementing 67 mM Sörensen’s buffer (final pH 7.2). The carbohydrate concentration in mFUM was 0.3% (w/v), and consisted of glucose for the first 16 h and from then on of a 1:1 (w/w) mixture of glucose and sucrose. Biofilms were incubated anaerobically at 37 °C for 64 h. Fresh medium was provided after 16 h and 40 h.

### 5.2. Treatments

In the present study, we used the nisin ZP (Handary SA, Brussels, Belgium) that is a high content form of nisin Z (>95% purity). The nisin ZP solution was prepared at a concentration of 200 μg/mL, as previous studies indicated that it showed bactericidal activity without cytotoxicity to human oral cells [7,15]. The mixture of eight D-AAs (D-methionine, D-leucine, D-tyrosine, D-tryptophan, D-serine, D-threonine, D-phenylalanine, and D-valine) (Sigma-Aldrich, St. Louis, MO, USA) was prepared as described previously [18,19]. All D-AAs were prepared at a concentration of 40 mM, except for D-tyrosine that was prepared at a concentration of 0.8 mM due to its low solubility. The 2% chlorhexidine (CHX) was prepared from a 20% stock CHX solution (Sigma Chemical, St. Louis, MO, USA). The final concentration of each component of the associations (nisin ZP + eight D-AAs and nisin ZP + 2% CHX) was the same as that of the agent alone.

After 64 h of biofilm growth, specimens were rinsed twice in phosphate buffered saline solution (PBS) and immersed in the following antimicrobial solutions: 2% CHX, a mixture of eight D-AAs, high-purity nisin ZP, high-purity nisin ZP + 2% CHX, and high-purity nisin ZP + eight D-AAs. The total treatment volume was 1 mL, and the treatment time was 5 min and 24 h. Three independent experiments were performed, including quadruplicates of each treatment and controls with no treatment. Three of the four discs were used to determine the total colony-forming units (CFUs), whereas one was randomly selected for confocal laser scanning microscopy (CLSM) analysis, as described below.

### 5.3. Bacterial Counts

After short- and long-term treatments, the biofilms were washed again in PBS before harvesting the biofilms for quantification. The hydroxyapatite disks were transferred to another tube containing 1 mL of 0.9% NaCl solution and vortexed vigorously for 3 min. Then, the disks were sonicated at 30 W in a Sonifier B-12 (Branson Ultrasonics, Danbury, CT, USA) for 10 s and vortexed for 30 s. Serial dilutions (10^−1^ to 10^−6^) were prepared in 0.9% NaCl and aliquots (50 μL) were spirally plated (Spiral System Model D, Spiral Systems Inc., Cincinnati, OH, USA) onto plates of Columbia blood agar supplemented with 5% whole human blood. The plates were incubated anaerobically at 37 °C for 5 d, and colony-forming units (CFUs) were counted with the aid of a stereomicroscope.

### 5.4. Vital Staining and CLSM Analysis

The biofilms were stained using LIVE/DEAD BacLight bacterial viability assay (Invitrogen, Zug, Switzerland) according to the instructions of the manufacturer. Stained biofilms were examined by CLSM at three randomly selected positions using a Leica TCS SP5 (Leica Microsystems, Heidelberg, Germany) as described previously [21]. Image acquisition was done in × 8 line average mode and scans were recombined and processed using IMARIS 7.6.5 software (Bitplane, Zurich, Switzerland).

### 5.5. Statistical Analysis

The null hypothesis that there was no microbiological difference between biofilms treated with different antibiofilm agents was tested by two-way ANOVA followed Tukey’s multiple comparisons test. A *p*-value < 0.05 was considered to indicate a significant difference. All statistical analyses were done with Prism v.8.4.3 statistical analysis software (GraphPad, La Jolla, CA, USA).

## Data Availability

Data is contained within the article; there is no supplementary material.
